# Posterior Scleritis as a Paraneoplastic Syndrome in Colon Cancer: A Case Report

**DOI:** 10.4274/tjo.galenos.2020.99836

**Published:** 2020-12-29

**Authors:** Dimitrios Kalogeropoulos, Konstantinos Katsikatsos, Michail Mitsis, Chris Kalogeropoulos

**Affiliations:** 1 University of Ioannina School of Health Sciences Faculty of Medicine, Department of Ophthalmology, Ioannina, Greece; 2 University of Ioannina School of Health Sciences Faculty of Medicine, Department of Surgery, Ioannina, Greece

**Keywords:** Scleritis, malignancy, colon cancer, paraneoplastic syndrome

## Abstract

This study presents a rare case of unilateral posterior scleritis as an ophthalmic manifestation of a paraneoplastic syndrome. A 61-year-old man presented to our department complaining of gradual worsening of vision in his left eye. Visual acuity was 10/10 and 3/10 in his right and left eye, respectively. He also mentioned that he experienced posterior ocular pain while sleeping at night, but was otherwise asymptomatic. His past ophthalmic and medical history were clear. A thorough clinical, imaging (fundus photography, optical coherence tomography, fluorescein angiography, and B-scan), and laboratory investigation was carried out. A diagnosis of posterior scleritis was made, but no obvious cause or underlying disease was identified even after a thorough systematic assessment. Regular follow-up within the next few months did not reveal any further pathological findings. Finally, 6 months after the initial presentation, the patient was diagnosed with colon cancer. Posterior scleritis can present as an ophthalmic manifestation of a paraneoplastic syndrome in patients with an underlying malignancy, even months before the presentation of systemic symptoms and diagnosis of the underlying disease. In conclusion, in patients (especially older adults) with posterior scleritis, the possibility of a malignant neoplasia must not be ignored or underestimated (paraneoplastic syndrome).

## Introduction

Scleritis is a painful, chronic, and potentially blinding inflammatory condition defined by edema and cellular infiltration of the entire thickness of the sclera. Non-infectious scleritis is the most common type and is frequently associated with an underlying systemic inflammatory condition of which it may be the first manifestation.^1^ Scleritis may be clinically isolated to the eye, but is frequently associated with a systemic disorder. Anatomically, it can be categorized into anterior and posterior. Posterior scleritis often appears in patients younger than 40 years old, who are usually otherwise healthy, but about one third of individuals over the age of 55 have an underlying systemic disease. Approximately 50% of cases are associated with a systemic disease, especially collagen disorders such as rheumatoid arthritis, Wegener granulomatosis, relapsing polychondritis, and polyarteritis nodosa.^2,3^ Some rare cases of malignant systemic disease have been described.^4,5^ Scleritis may be the first or only presenting clinical manifestation of these severe and potentially lethal clinical entities. An early and accurate diagnosis of the associated systemic or infectious etiology in combination with appropriate treatment can stop the relentless progression of both ocular and systemic processes.^6^ The diagnostic approach to scleritis can be challenging due to its perplexing and varied clinical signs and symptoms.^3^ Herein, we report a case of posterior scleritis as the initial and only manifestation of paraneoplastic syndrome in a patient with colon cancer.

## Case Report

A 61-year-old Caucasian man presented with a 2-month history of gradual decline in visual acuity (VA) and moderate pain in the left eye that radiated to the orbit and was awakening him at night. He reported no other ocular or systemic symptoms. His past ophthalmic history was clear and his blood pressure was well-controlled with antihypertensive medication. Otherwise, his past medical history was unremarkable, without any evidence of musculoskeletal diseases or systematic vasculitis.

**Figure 1:**
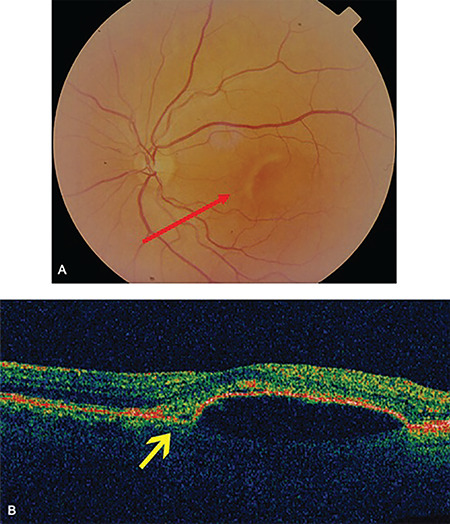
Posterior scleritis. A) Left eye posterior pole showing retinal elevation along with a retinal pigment epithelium (RPE) rip (red arrow). B) Optical coherence tomography of the left eye showing sub-RPE fluid leading to RPE detachment. The area corresponding to the RPE rip is indicated by the yellow arrow

**Figure 2:**
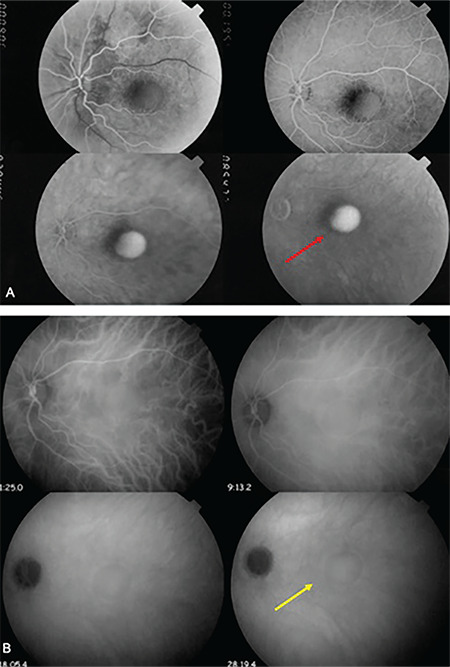
Posterior scleritis. A) Fluorescein angiography showing pooling of fluorescein without leakage (red arrow). The adjacent dark area corresponds to the RPE-rip. B) Indocyanine green angiography showing hypercyanescent area corresponding to the sub-RPE fluid accumulation (yellow arrow)

**Figure 3:**
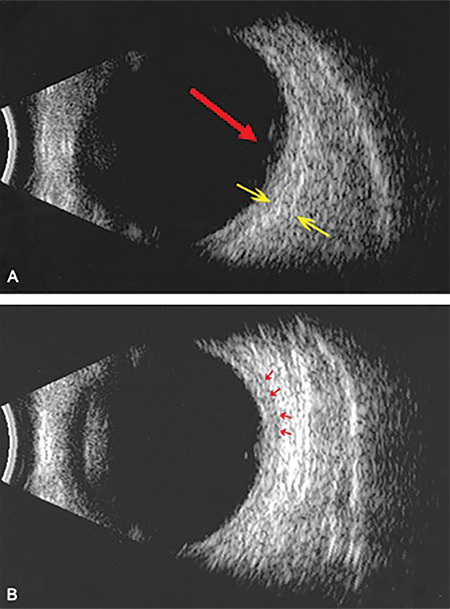
Posterior scleritis: B-Mode echography. A) Retinal elevation due to sub-RPE fluid accumulation (red arrow) with thickened sclera (yellow arrows). B) Mild choroidal thickening with discrete fluid in sub-Tenon’s space (red arrows) RPE: Retinal pigment epithelium

On presentation, his Snellen VA was 10/10 (uncorrected) in the right eye (OD) and 3/10 (best corrected VA) in the left eye (OS). A thorough clinical, imaging, and laboratory investigation was carried out. Slit-lamp biomicroscopy of the anterior chamber revealed that there was no presence of flare and cells. Pupillary reflex and eye movements were normal in both eyes. Fundoscopy showed a retinal elevation at the posterior pole of OS along with a retinal pigment epithelium (RPE) rip (Figure 1a). The RPE rip probably occurred as a result of inflammation and exudation of fluid causing pressure on the RPE.^7^ Optical coherence tomography scan (Figure 1b) confirmed the presence of sub-RPE fluid leading to a RPE detachment. Fluorescein angiography demonstrated subretinal pooling of fluorescence without leakage in the same area the retinal elevation was detected (Figure 2a). On the other hand, indocyanine green angiography  showed a hypercyanescent area corresponding to the sub-RPE accumulation (Figure 2b). The sub-RPE fluid was also verified by B-mode ultrasound scan (Figure 3a, b). More specifically, ultrasound imaging highlighted retinal elevation (due to sub-RPE fluid accumulation) with thickened sclera and mild choroidal thickening with discrete fluid in sub-Tenon’s space. Our diagnostic work-up aimed to exclude infectious or autoimmune causes of scleritis. Results of laboratory investigations, including full blood count and biochemistry assays, were unremarkable. Serum rheumatoid factor, angiotensin-converting enzyme, antinuclear antibodies, antineutrophilic cytoplasmic antibodies (p and c), enzyme-linked immunosorbent assay for human immunodeficiency virus (1 and 2), serology for syphilis (Treponema pallidum hemagglutinin antigen, Venereal Disease Research Laboratory) and viral hepatitis (B and C) were negative. Erythrocyte sedimentation rate and C-reactive protein were within normal limits. Furthermore, a routine systematic assessment did not raise any suspicion about the possibility of an underlying disease.

Taking into account the presenting symptoms together with the aforementioned findings, a diagnosis of posterior scleritis was made and oral nonsteroidal anti-inflammatory agents were administered for symptomatic relief. However, at that time it could not be associated with any obvious cause or known systemic disease. Regular follow-up over the next few months did not reveal any further pathological findings. Finally, 6 months after the initial presentation, the patient was diagnosed with colon cancer. The colon carcinoma was asymptomatic and diagnosed during a routine examination when a baseline colonoscopy was performed. At that time, all the required investigations were performed at the department of surgery in order to establish the diagnosis. A previous colonoscopy in his early 50s had not revealed any significant findings.

## Discussion

Scleritis is an infrequent ocular inflammatory entity. The majority of ophthalmologists may not encounter more than 2 cases of scleritis per year.^8^ It can lead to potentially severe ocular complications and approximately 50% of cases are associated with topical or systemic diseases, some of which may have lethal consequences.^3^ Some of the most common and well-described systemic diseases associated with scleritis are systemic lupus erythematosus, inflammatory bowel disease, relapsing polychondritis, rheumatoid arthritis, polyarteritis nodosa and granulomatosis with polyangiitis (formerly called Wegener’s).^3^ Infectious agents such as herpes simplex viruses, tuberculosis, *Pseudomonas*, and *Aspergillus* may cause severe and difficult to treat scleritis.^8^ Albeit rare, scleritis may be the initial or only feature of a masquerade^9,10^ or paraneoplastic syndrome.^4^ Therefore, malignancies must always be included in the differential diagnosis in cases of scleritis with no obvious cause.

Masquerade syndromes are typically described as pathologies that mimic inflammatory clinical entities but which are associated with a neoplastic process. Detailed medical history and thorough clinical assessment together with specific laboratory and histopathologic investigations can help establish an accurate diagnosis. A wide spectrum of conditions may lead to features imitating an inflammatory condition.^11^ On the other hand, paraneoplastic syndromes involve complications of a systemic malignancy that present as various disorders of one or more systems, including dermatological, endocrine, hematological, neuromuscular, or even ocular abnormalities. Paraneoplastic syndromes are defined by a rapid development of atypical signs and symptoms without any obvious etiology or features of metastasis and may manifest up to 2 years before a diagnosis of cancer. Pathogenetic mechanisms encompass cell-mediated and humoral immune responses against antigens expressed by malignant cells, leading to inflammation and cellular destruction.^12^ More specifically, following the activation of host immune mechanisms, antibodies are produced against the cancer antigen, resulting in autoimmunization and the production of autoantibodies against normal host tissue.^13,14^ Any type of cancer may be associated with a paraneoplastic syndrome. Small cell lung cancer, non-small cell lung cancer, melanoma, and cancers of the breast, uterine, and thyroid are the most common cancers associated with paraneoplastic syndrome.^11^ Cancer-associated retinopathy, optic neuropathy, bilateral diffuse uveal melanocytic proliferation, Lambert-Eaton myasthenic syndrome, and melanoma-associated retinopathy are some of the noted ophthalmic conditions that accompany paraneoplastic syndrome with ocular manifestations.^11^ Early diagnosis of a paraneoplastic syndrome is vital for detecting the underlying disease and eventually facilitating the appropriate treatment and follow-up.^12^

Posterior scleritis is usually associated with a systemic disease (infectious or autoimmune). In approximately 2 out of 3 cases with posterior scleritis, an underlying disease is not revealed. According to the current literature, several masquerade symptoms, such as lymphomas, can manifest with scleritis.^10,15,16^ For instance, Hoang-Xuan et al.^10^ reported a new masquerade syndrome presenting with features of mucosal-associated lymphoid tissue lymphoma associated with choroidal white dots and scleritis. Similarly, Mohsenin et al.^16^ described the case of a 53-year-old man with coexisting necrobiotic xanthogranuloma and chronic lymphocytic leukemia presenting with scleritis and uveitis. In both of these cases, scleritis occurred as a masquerade syndrome. To our knowledge, although malignant neoplasias can masquerade as posterior scleritis,^17,18,19^ there is only one recorded case of scleritis presenting in the context of a paraneoplastic syndrome.^4^ Therefore, our case is unique because posterior scleritis as a paraneoplastic syndrome has not been described until now. In particular, there are no available reports of patients with colon cancer developing posterior scleritis.

Individuals with scleritis must be evaluated by means of a detailed medical history, ocular performance, and general physical examination, as well as appropriate laboratory and imaging investigations. A correct and rapid diagnosis of scleritis can halt the progression of topical and systemic disease, thus preventing destruction of the globe while prolonging survival and improving quality of life. In patients with posterior scleritis, especially older adults, the possibility of paraneoplastic syndrome due to a malignant neoplasia must not be ignored or underestimated.
